# Identification of *TSGA10* and *GGNBP2* splicing variants in 5′ untranslated region with distinct expression profiles in brain tumor samples

**DOI:** 10.3389/fonc.2023.1075638

**Published:** 2023-02-13

**Authors:** Reihane Kazerani, Pouya Salehipour, Mohammadreza Shah Mohammadi, Elnaz Amanzadeh Jajin, Mohammad Hossein Modarressi

**Affiliations:** ^1^ Department of Biology, Science and Research Branch, Islamic Azad University, Tehran, Iran; ^2^ Department of Medical Genetics, School of Medicine, Tehran University of Medical Science, Tehran, Iran; ^3^ Functional Neurosurgery Research Center, Shohada Tajrish Comprehensive Neurosurgical Center of Excellence, Shahid Beheshti University of Medical Sciences, Tehran, Iran

**Keywords:** cancer testis antigens (CTA), testis specific gene antigen 10 (TSGA10), gametogenetin binding protein 2 (GGNBP2), 5’ untranslated region, brain tumors, alternative splicing (AS)

## Abstract

**Introduction:**

Brain tumors (BTs) are perceived as one of the most common malignancies among children. The specific regulation of each gene can play a critical role in cancer progression. The present study aimed to determine the transcripts of the *TSGA10* and *GGNBP2* genes, considering the alternative 5′UTR region, and investigating the expression of these different transcripts in BTs.

**Material and methods:**

Public data on brain tumor microarray datasets in GEO were analyzed with R software to evaluate the expression levels of *TSGA10* and *GGNBP2* genes (the Pheatmap package in R was also used to plot DEGs in a heat map). In addition, to validate our in-silico data analysis, RT-PCR was performed to determine the splicing variants of *TSGA10* and *GGNBP2* genes in testis and brain tumor samples. The expression levels of splice variants of these genes were analyzed in 30 brain tumor samples and two testicular tissue samples as a positive control.

**Results:**

In silico results show that the differential expression levels of *TSGA10* and *GGNBP2* were significant in the GEO datasets of BTs compared to normal samples (with adjusted p-value<0.05 and log fold change > 1). This study’s experimental results showed that the *TSGA10* gene produces four different transcripts with two distinct promoter regions and splicing exon 4. The relative mRNA expression of transcripts without exon 4 was higher than transcripts with exon 4 in BT samples (p-value<001). In *GGNBP2*, exon 2 in the 5′UTR region and exon 6 in the coding sequence were spliced. The expression analysis results showed that the relative mRNA expression of transcript variants without exon 2 was higher than other transcript variants with exon 2 in BT samples (p-value<001).

**Conclusion:**

The decreased expression levels of transcripts with longer 5′UTR in BT samples than in testicular or low-grade brain tumor samples may decrease their translation efficiency. Therefore, decreased amounts of TSGA10 and GGNBP2 as potential tumor suppressor proteins, especially in high-grade brain tumors, may cause cancer development by angiogenesis and metastasis.

## Introduction

Primary brain tumors (BTs) originate from various types of nerve cells (1). Nerve cell neoplasms are regarded as one of the most prevalent tumors in children aged 0-14 years old and are one of the leading causes of death among this age group. These tumors are often associated with many clinical symptoms, such as headache, nausea, vomiting, vision loss, and others. Multiform glioblastoma is one of the most common and lethal primary brain tumors. The survival rate of these patients varies according to the histology and molecular characteristics of the tumor (2, 3). Different genetic mutations have helped to differentiate BTs, their classification, prognosis, and therapeutic responses (4–7). Moreover, the expression alterations of the oncogene or tumor suppressor gene can lead to tumor progression (8) (1).

Cancer testis antigens (CTAs) are normally and primarily expressed in germinal cells such as the testis and fetal ovary, along with the trophoblasts of the placenta under homeostatic conditions. They can be expressed in multiple malignancies (9). Given this, two types of CTAs are detected: encoded on the X chromosome (cancer testis-X antigens) and not encoded on the X chromosome (non-X cancer-testis antigens). The CT-X genes are mainly expressed in spermatogonia, while non-X CT genes are expressed in the later stage of germ-cell differentiation (10, 11). The expressions of CTAs in cancers supply prognostic value. Since CTAs are highly immunogenic and have limited expression in normal tissues, they became extremely appropriate targets for immunotherapy. However, the heterogeneous expression of many CTAs impedes their prosperous use in the clinical approach (12). Germ cell development processes and tumor progression are remarkably similar in terms of meiosis inducing, immortalization, invasion, and migration (10), indicating the pivotal role of testis-specific genes in cancer studies.

Testis-specific gene antigen 10 (*TSGA10*) is one of the potential CT-antigens (Cancer/Testis Antigen 79), predominantly expressed in testis and overexpressed in some tumors, such as BTs (13, 14). The overexpression of TSGA10 has been observed in embryogenesis, brain development, and some malignancies, including BTs. *TSGA10* was discovered in 2001, and the protein consists of two fragments, including an N-terminal, 27 kDa, located in the fibrous sheath of the sperm tail, and a C-terminal, 55 kDa (14–16). The accumulation of TSGA10 inhibited the hypoxia-inducible factor 1a (HIF-1a) and prevented angiogenesis and metastasis (17). Since TSGA10 is involved in neuronal development and BTs, it could potentially serve as a biomarker for diagnostic and therapeutic targets (13).

Another important potential CT-antigen is Gametogenetin binding protein 2 (GGNBP2), located on chromosome 17q12, and is known as DIF3, LZK1, DIF-3, LCRG1, ZFP403, and ZNF403 (18). GGNBP2 is associated with spermatogenesis and is mainly expressed in the late stage of spermatocytes and the early stage of round spermatids, especially in the initial stage of spermatid meiosis (19). This gene acts as a tumor suppressor in some cancers, such as breast cancer, glioma, prostate cancer, and ovarian cancer (20–22). Its downregulation in primary laryngeal carcinomas leads to laryngeal tumorigenesis (23).

Alternative splicing is a key to transcriptome and proteome diversification in eukaryotes (24, 25), resulting in the expression of multiple RNA and vast protein diversity from one gene (26). A defect in this mechanism induces many diseases, including cancers. Cancer cells perform significant transcriptome alteration partly by cancer-specific splicing isoforms. It is identified as an essential indication for tumor progression and therapy in cancer (25, 27, 28). 5′UTR plays a critical role in regulating gene expression and controlling mRNA translation and stability (29). Various 5′UTR sequences derived from alternative splicing of non-coding exons can give rise to diverse variants of mature mRNAs (24, 29).

Untranslated regions contain exons around the coding sequence, which are transcribed into mRNA but are not translated into protein. These areas, named 5′UTR and 3′UTR, play significant roles in translational regulation. The average length of a 5′UTR is 100bp to 220bp among different species (30). The minimum length of 5′UTR is 18 nucleotides and the longest identified 5′UTR in humans is an oncogene mRNA with 2858 nucleotides (31). In vertebrates, transcription factors, proto-oncogenes, tumor suppressors, growth factors, and their receptors seek to manufacture transcripts with longer and more complicated 5′UTR regions (32, 33).

This study analyzed the 5′UTR region of *TSGA10* and *GGNBP2*, considering the significance of the regulatory effects of 5′UTR and unclear mechanisms regulating the expression of *TSGA10* and *GGNBP2* in normal and cancer cells. Given the roles of *TSGA10* and *GGNBP2*, cells may regulate the expression of these genes in both the transcription and translation stages. This article reports on the different expression patterns of *TSGA10* and *GGNBP2* transcript variants with different 5′UTR sequences in human brain tumor samples. Moreover, we investigated the pathways and genes related to *TSGA10* and the brain tumor.

## Materials and methods

### Patients and samples

Thirty BT tissue samples belonging to the Shohadaye Tajrish Hospital from May 2018 to February 2019 were obtained according to the protocols of the Medical Ethics Committee. The tissues were collected in tubes and treated with RNA later. All the patients suffered from primary BTs with no radiotherapy or chemotherapy medical history. The patients’ characteristics, tumor types, and tumor grades were recorded for all patients (**Table 1**). Then, two normal testis tissues were derived from the prostate cancer patients treated with orchiectomy. Written informed consent was obtained from the participants.

**Table 1:** Demographic and pathological characteristics of the study participants.

Parameter	Brain tumors samples
Patient number	30
Marital status	
Married	15 (50%)
Single	15 (50%)
Gender	
Female	17 (56.66%)
Male	13 (43.33%)
Age at diagnosis (Years)	
<15	23 (76.66%)
≥15	7 (23.33%)
Pathologic Diagnosis	
Schwannoma	3 (10%)
Astrocytoma	13 (26.66%)
GBM	9 (16.66%)
Medulloblastoma	5 (10%)
Grade	
I	3 (13.33%)
II	13 (60%)
III	–
IV	14 (26.66%)
Tumor size (cm)	
<3cm	7 (23.33%)
≥3cm	23(76.66%)
Smoking	
Yes	9 (30%)
No	21 (70%)

### Bioinformatics analysis of target genes

We searched for public data on BT microarray datasets in order to assess the expression levels of *TSGA10* and *GGNBP2*. The GEO (34) database was searched by applying filters to find the experiments, including healthy brain samples and intact brain samples with different BTs. Accordingly, all the studies or samples with BT patients undergoing any treatment methods were removed from the data. Datasets with GSE15824 (35), GSE35493 (36), and GSE50161 (37) were finally selected to be included in this study. Matrix data and description data files were downloaded to find differentially expressed genes (DEGs) and the screening expression levels of *TSGA10* and *GGNBP2*. Given that each experiment included various types of tumors, we selected the samples required for each comparison. In this regard, a comparison of glioblastoma (12 samples) vs. healthy control (9 samples) and medulloblastoma (21 samples) vs. healthy control (9 samples) was conducted from the GSE35493 dataset. Moreover, BTs (33 samples including glioblastoma and medulloblastoma samples) vs. healthy control (12 samples) were compared from this dataset. Furthermore, Oligodendrioma (7 samples) vs. healthy control (2 samples), glioblastoma (25 samples) vs. healthy control (2 samples), and astrocytoma (11 samples) vs. healthy control (2 samples) were compared from the GSE15824 dataset. A comparison of BTs (42 samples including mentioned BT samples) vs. healthy control (2 samples) was also performed for this platform. Then astrocytoma (15 samples) vs. healthy control (13 samples), ependymoma (46 samples) vs. healthy control (13 samples), glioblastoma (34 samples) vs. healthy control (13 samples), and medulloblastoma (22 samples) vs. control (13 samples) were compared from the GSE50161 dataset. Finally, BTs (117 samples including mentioned BT samples) and healthy control (13 samples) were compared from the same dataset.

### Data analysis and DEGs identification

Analyses were performed in R, using Limma (38), GEOquery (39), and UMAP (40) packages. The raw data of the microarray was preprocessed to convert probes into gene symbols, using GEOquery software. Then, data clean-up was performed to remove probes with no gene symbols or gene symbols with more than one probe using the UMAP package. Data normalization and DEG screening between BTs and healthy controls were performed in R, using the Limma package (version 4.1). The significant differential expression level was set at adjusted p-value<0.05 and log-fold change > 1. The pheatmap package in R was also used to plot DEGs as a heat map (41).

### Primer design

**Figure 1:**
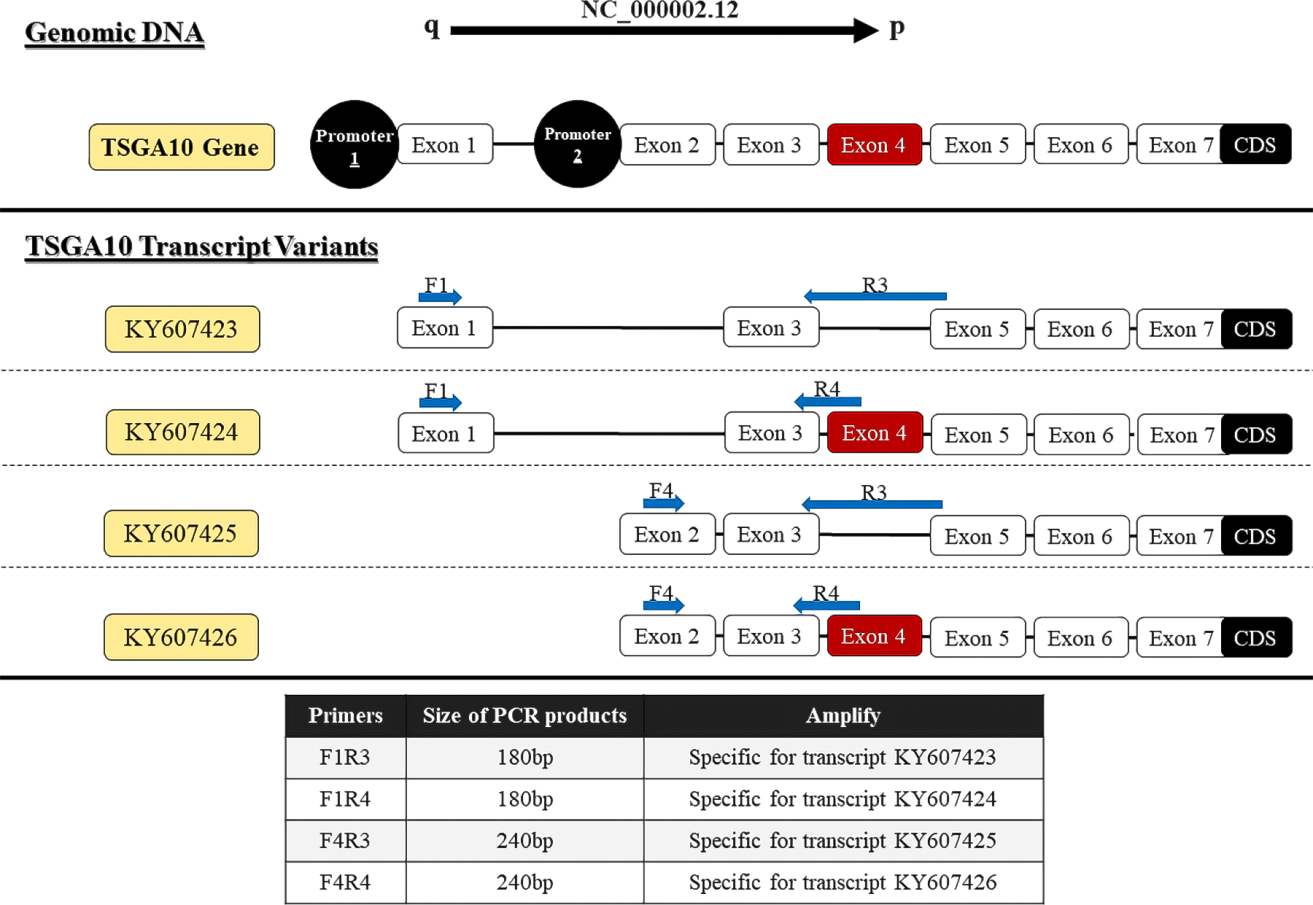
Locations of primers to amplify TSGA10 splice variants. Exons number 1-7, which form the 5′UTR region of four experimentally approved transcript variants. Primers were designed to detect spliced variants based on exon 4 splicing and two distinct promoters (shown in red).

**Figure 2:**
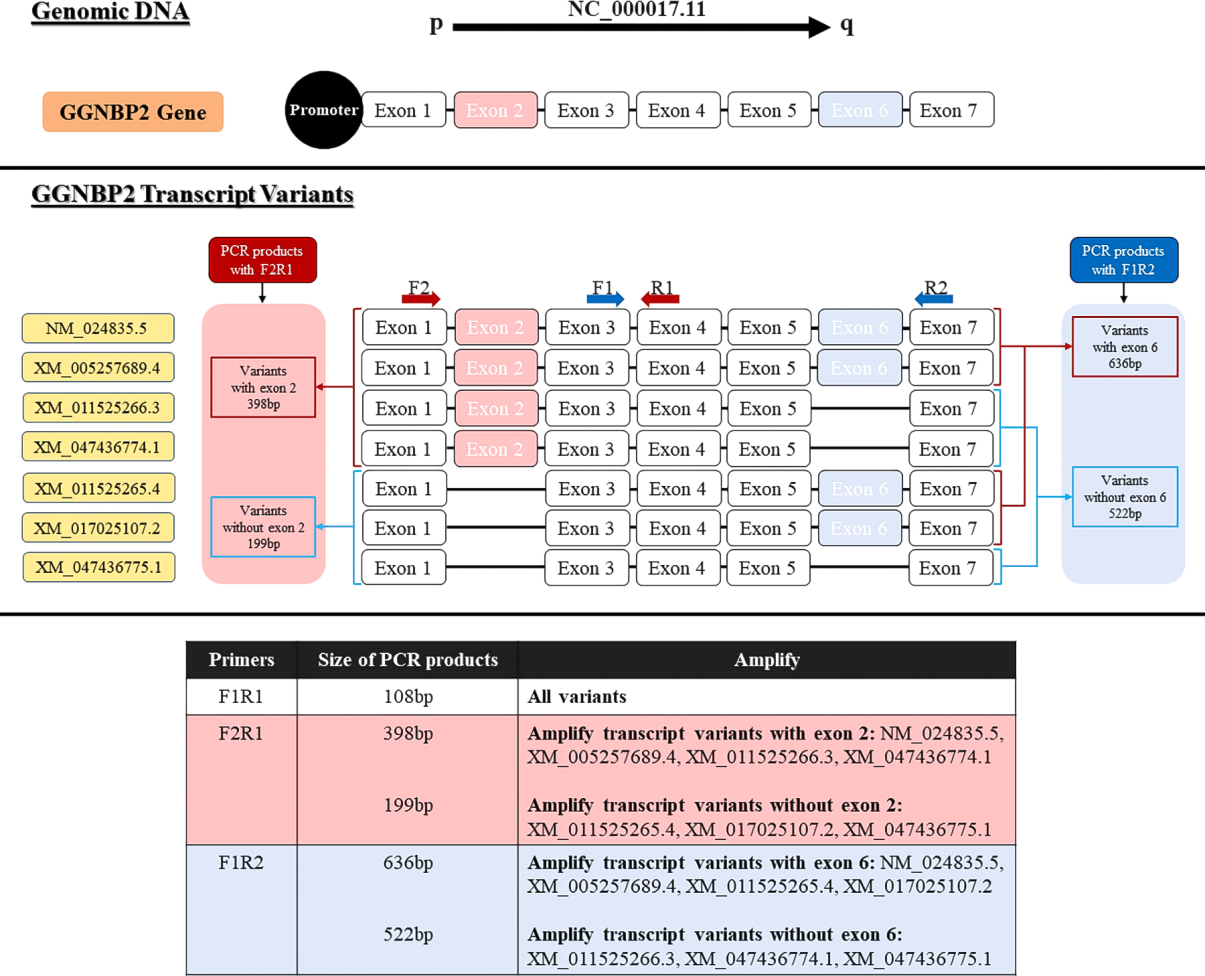
Locations of primers to amplify GGNBP2 splice variants. Exons number 1-7 show the GGNPB2 gene and 7 different transcript variants of this gene. Primer pair F2R1 were designed to detect spliced variants based on exon 2 splicing (shown in pink) and primer pair F1R2 were designed to detect spliced variants based on exon 4 splicing (shown in blue).

The primer sequence of *TSGA10* gene transcripts for the 5’UTR region and one reverse primer from the coding sequence of *TSGA10* were designed to check the expressed transcripts from the coding region (**Figure 1**). The primers were used from Salehipour et al.’s study to examine the transcripts of this gene in breast cancer (42). NCBI Primer-Blast was applied to design the primers of the *GGNBP2* gene for alternative splicing analyses, and their specificity was checked (**Figure 2**). Then, the primers designed by Gene Runner software were examined. A pair of primers for housekeeping gene phosphoglucomutase 1 (PGM1) was designed to control cDNA synthesis from our RNA samples. **Table 2** shows the sequence of the primers.

**Table 2:** DNA oligonucleotides used in this study (F: Forward primer and R: Reverse primer).

Primer	Sequence (5’-3’)
TSGA10	
**F6**	ACAGAGCGCTTTGAAAGGGAG
**R6**	AGATCGATGGTGAGCACGTT
**F1**	AGCACAGAGATAACGGCCAG
**F4**	TGTAAGGCAGGCAGGTAGAC
**R3**	TGTCTGTTTGAGATCTTCAATCTGT
**R4**	CACAAAGGTATCCAAATGCCTGT
GGNBP2	
**F1**	CATCAGAATAATGGTGCACAGC
**F2**	CGACCACGAAAACGGTGAAG
**R1**	ACTCAGGACTTCGCGTGAT
**R2**	TCGTTCATGTGGACAGCACC
Housekeeping gene	
**PGM-F**	AGCATTCCGTATTTCCAGCAG
**PGM-R**	GCCAGTTGGGGTCTCATACAAA

### Total RNA extraction

Trizol (Invitrogen) was used to extract total RNA from 50mg tissue samples according to the manufacturer’s instructions. The quantity (on average 700 ng in a total volume of 30ml) and purity (average A260/A280 ratio 1.7) of each RNA sample were measured by NanoDropND-2000 Spectrophotometer (Thermo Fisher Scientific, Wilmington, DE), and their integrity was confirmed by electrophoresis on 1% agarose gel (UltraPure TM Agarose; Merk) in 2.2M formaldehyde. The high-quality RNA samples with no degradation (in which a smear was observed with two bands, S18 and S28, corresponding to ribosomal RNA, indicating the appropriate quality of RNA for cDNA production) were stored at -80°C for a maximum of one month before further analyses.

### Two-step RT-PCR

The first strand of cDNA was synthesized using a cDNA synthesis kit (Thermo Fisher Scientific). Then 2000ng of total RNA from the testis and BT samples were reversely transcribed using random hexamer and oligo-dT primers. The PCR assay with a specific primer for PGM1 was carried out to verify the existence of cDNA (120bp band). The cDNAs were used for the PCR assays with primer pairs F1R3, F1R4, F4R3, and F4R4 to identify the expression of each *TSGA10* transcript variant in the testis and BT samples specifically. The other PCR assays with primer pairs F1R1, F1R2, and F2R1 were used to identify the expressed transcript variants of *GGNBP2* and exon splicing in the testis and BTs. The PCR assays were performed under initial heating for 5 minutes at 94°C. In the next phase, 30 thirty-second cycles of amplification were performed at 94, 56, and 72°C, followed by a final extension of 5 minutes at 72°C, after which the PCR products were separated on 2% agarose gel and visualized under UV light after DNA staining. The ImageJ program was employed to quantify the bands in a range of 0-4.

### Statistical analysis

The *PGM1* mRNA levels were applied to normalize the mRNA levels of different *TSGA10* and *GGNBP1* transcript variants, with relative mRNA levels as the ratios between *TSGA10* and *GGNBP2* transcript variants and *PGM*. Statistical analyses were conducted using IBM SPSS software. The expression of different *TSGA10* and *GGNBP2* transcript variants in the BT tissues was compared using the Wilcoxon test. The correlation between the expression of *TSGA10* and *GGNBP2* transcript variants with the clinic pathological variables was examined through the nonparametric Mann-Whitney U test. A two-tailed p-value <0.05 was set as the significance level.

## Results

### Data profile of brain tumor GEO datasets

The microarray data in the GSE15824 (**Figure S1**) were divided into four groups (namely GBM, Astrocytoma, Oligodandrocytoma, and control). Those in GSE35493 (**Figure S2**) were divided into two groups (namely BTs and control), and those in GSE50161 (**Figure S3**) were separated into five groups (namely GBM, Astrocytoma, ependymoma, medulloblastoma, and control). Each of these datasets was analyzed separately with R software. Accordingly, each sample of the BT groups was compared with the control sample of each dataset individually.

### Differential expression analysis of *TSGA10* and *GGNBP2*

**Figure 3:**
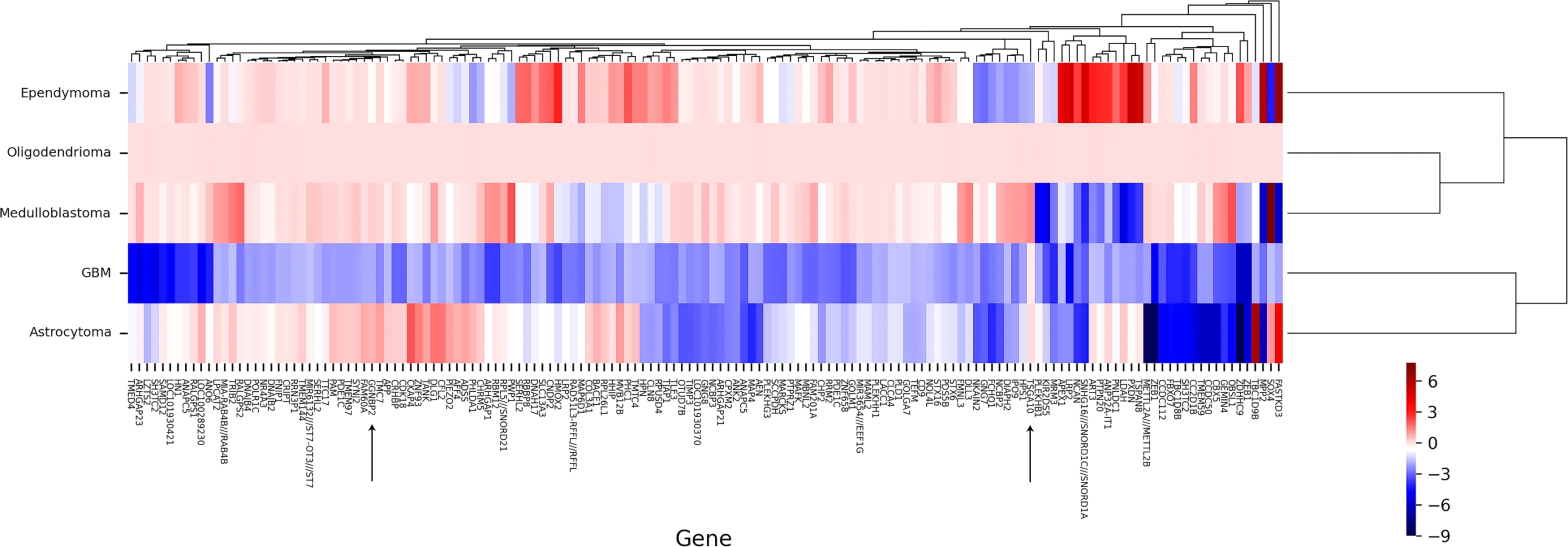
Clustering heat map of DEGs by normal tissues (N) and cancer tissues (C) in BTs. A representative heat map of gene expression was obtained from microarray analyses. Heat map of DEGs in a GMB, astrocytoma, medulloblastoma, oligodendrioma, and ependimoma is shown to express targeting genes *TSGA10* and *GGNBP2*. In general, these two genes show upregulation in all BTs, except for *GGNBP2* in GBM and *TSGA10* in ependymoma, representing downregulation. Each column represents one type of BT, and each lane represents one gene. Gene expression levels are represented by a color scale ranging from red to blue, according to the log2fold change of genes. Accordingly, the redder the log2 fold change, the more upregulation it has, and the higher the level of expression obtained, meaning it goes towards blue, and the more its expression decreases. Heat maps of DEGs from two microarray datasets were generated with R software.

For data precision and consistency, having used the UMAP package in R software to reduce the dimension of the data, we then adjusted the p-value to modify the data Adjusted p-value under 0.05 was set as the significance level. In the GSE15824 dataset, the *TSGA10* expression was reduced in the GBM tumor with a log2 fold change of -2.59 and increased in the oligodendrocytoma tumor with a log2 fold change of 2.521. In the GSE50161, the *TSGA10* expression was also reduced in the ependymoma tumor with a log2 fold change of -2.67. The expression of GGNBP2 was significantly upregulated only in the GSE35493 dataset and in GMB tumor samples with a log2 fold change of 0.88 (**Table 3**). **Tables S1**, **S2** present changed expression of the TSGA10 and GGNBP2 genes with the exact p-value and log FC. **Figure 3** illustrates the heat map of the differential expression of the genes. The results showed that in GBM tumor samples, being regarded as the hardest tumor among brain tumors, the expression of many genes was downregulated.

**Table 3:** Results of DEG analysis for comparison between brain tumors and control samples.

Dataset	Comparison	GGNBP2		TSGA10	
		log2 fold change	Adjusted p-value	log2 fold change	Adjusted p-value
**GSE15824**	GBM vs Control	-0.523	0.568	-2.595	0.032
	Astrocytoma vs Con	0.295	0.054	-0.629	0.058
	Oligodendrocytoma vs Con	-0.417	0.493	2.521	0.001
**GSE35493**	Medulloblastoma vs Con	-0.429	0.409	-0.202	0.571
	GBM vs Con	0.881	0.011	0.235	0.359
**GSE50161**	Astrocytoma vs Com	-0.044	0.914	0.226	0.474
	Ependymoma vs Con	-0.046	0.906	-2.677	1.48E-04
	GBM vs Con	0.387	0.270	-0.144	0.742
	Medulloblastoma vs Con	-0.075	0.888	-0.137	0.724

### Expressed transcript variants of *TSGA10* in brain tumor

**Figure 4:**
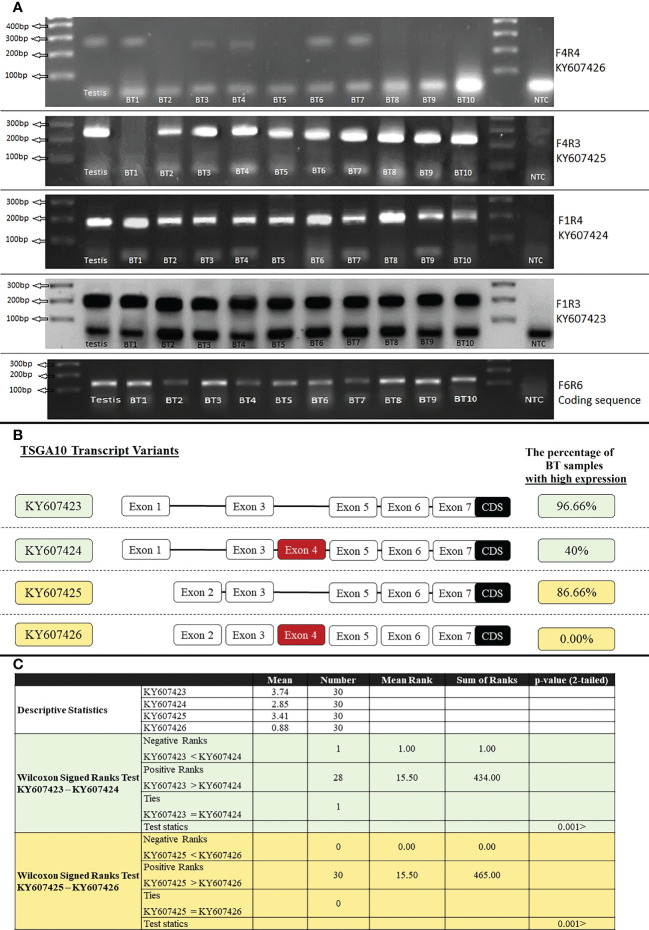
RT-PCR amplification of *TSGA10* transcript variants. **(A)**. 1500ng of total RNA from Testis and Brain tumor (BT) samples were reversely transcribed and amplified by specific primers for *TSGA10* transcript variants. Primer pairs F4R4, F4R3, F1R4, and F1R3 were used to amplify transcript variants KY607426 (240bp), KY607425 (240bp), KY607424 (180bp), and KY607423 (180bp), respectively. Primer pair F6R6 amplifies all transcript variants of the *TSGA10* gene (amplify coding sequence region) and produces a 120bp band. Lane NTC is a negative control. Bands were separated on 2% agarose gel. **(B)**. Relative mRNA expression was calculated based on the ImageJ program and a relative expression higher than 3 was considered a high expression. Percentage of BT samples with high expression were calculated and shown for each spliced variant of the TSGA10 gene. **(C)**. Non-parametric statistical Wilcoxon Signed Ranks Test were used to compare KY607423 vs KY607424 and KY607425 vs KY607426 transcript variants in BT samples. The results were shown that KY607423 and KY607425 transcripts (which do not have exon 4) have higher relative mRNA expression than KY607424 and KY607426 transcripts (which have exon 4), respectively in 30 BT samples (P <.001).

Primer F6R6 binds to the coding sequence region (135bp), and the expression of this region was observed in all tissue samples of BT patients. The expression of various *TSGA10* gene transcript variants was noticed in all BT samples (30 samples) regardless of their grade and stage. Then, specific primers were used for RT-PCR amplification of *TSGA10* transcript variants, and the ImageJ program calculated the relative mRNA expression. Both promoters were active, producing *TSGA10* mRNAs; however, the BT tissue samples witnessed a decreased expression of the transcript variants containing exon 4. The relative mRNA expressions were divided into two groups: high expression with relative mRNA expression higher than 3 and low expression with relative mRNA expression lower than 3. The percentage of samples with high expression of transcript variants without exon 4, including KY607423 and KY607425, was 96.66% (mean of relative mRNA expression was 3.74) and 86.66% (mean was 3.41), respectively. The percentage of samples with high expression of transcript variants containing exon 4, including KY607424 and KY607426, was 40% (mean of relative mRNA expression was 2.85) and 0.00% (mean was 0.88), respectively (**Figure 4**). The expression analysis results for the BT samples showed that the relative mRNA expression of transcripts without exon 4 (with a shorter 5′UTR sequence) was higher than transcripts with exon 4 in BT samples (p-value<001). There was no significant difference between Low (Stage I + II) and Advanced (Stage III + IV) regarding the separation of stage expression.

### Expressed transcripts of the human *GGNBP2* gene

**Figure 5:**
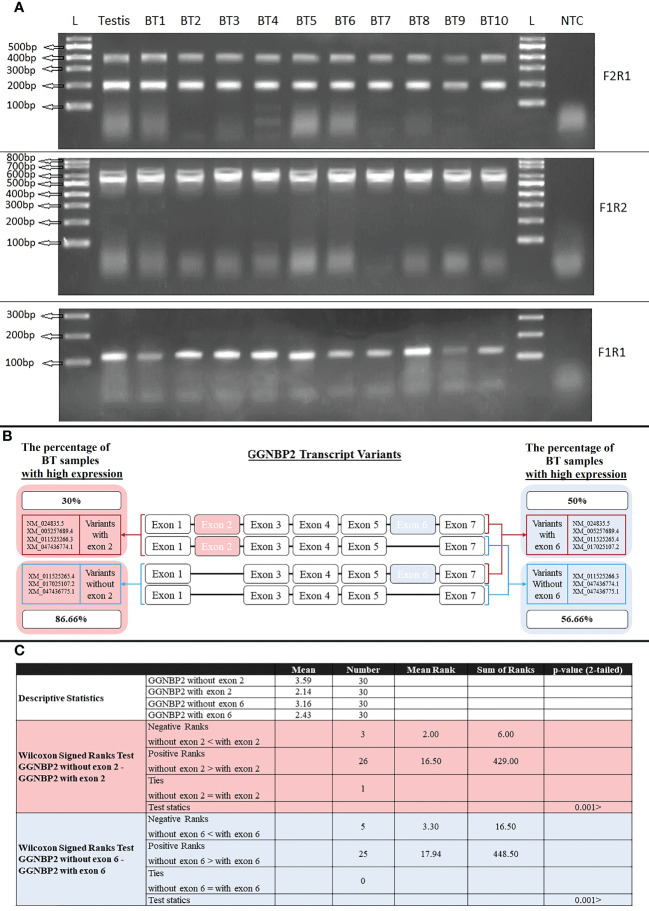
RT-PCR amplification of *GGNBP2* mRNAs containing different splicing variants. **(A)**. RT-PCR amplifications from Testis and BT samples were performed using the following combinations of primers: Primer pair F2R1 produced 398bp and 199bp bands indicating the exon 2 splicing of the *GGNBP2* gene. Primer pair F1R2 produced 636bp and 522bp bands indicating the exon 6 splicing of the *GGNBP2* gene. Primer pair F1R1 amplifies all transcript variants of *GGNBP2* and produces a 108bp band. Lane NTC is a negative control. Lane L is a 100bp DNA ladder. Bands were separated on 2% agarose gel. **(B)**. Relative mRNA expression was calculated based on the ImageJ program and a relative expression higher than 3 was considered a high expression. Percentage of BT samples with high expression were calculated and shown for each spliced variant of the *GGNBP2* gene. **(C)**. Non-parametric statistical Wilcoxon Signed Ranks Test were used to compare *GGNBP2* exon 2 and exon 6 spliced variants. The results showed that spliced variants without exon 2 and exon 6 have higher relative mRNA expression than spliced variants with exon 2 and exon 6, respectively (P <.001).

Seven transcript variants of the *GGNBP2* gene with different 5′UTR regions and coding sequences were expressed in human testicular tissue samples. The two essential splicing exons were located upstream of this gene, including the splicing of exon 2 in the 5’UTR region and the splicing of exon 6 in the coding region. The expression of *GGNBP2* transcript variants was investigated in all BT samples (30 samples) according to the splicing of exons 2 and 6. The difference in expression of transcript variants spliced in exons 2 was investigated using the primer F2R1. The relative mRNA expressions were divided into two groups: high expression with relative mRNA expression higher than 3 and low expression with relative mRNA expression lower than 3. The percentage of samples with a high expression of transcript variants without exon 2 (amplified with primer F2R1, 199bp) and with exon 2 (amplified with primer F2R1, 398bp) was 86.66% (the mean of relative mRNA expression was 3.59) and 30% (mean was 2.14), respectively. Moreover, the difference in expression of transcript variants spliced in exon 6 was investigated using the primer F1R2. The percentage of high expression transcript variants without exon 6 (amplified with primer F1R2, 522bp) and with exon 6 (amplified with primer F1R2, 636bp) were 56.66% (mean of relative mRNA expression was 3.16) and 50% (mean was 2.43), respectively (**Figure 5**). The results indicated that the relative mRNA expression of transcripts without exon 2 (with a shorter 5′UTR sequence) was higher than transcripts with exon 2 in BT samples (p-value<001). There was no significant difference between Low (Stage I + II) and Advanced (Stage III + IV) brain tumors, considering the expression levels of the transcripts.

## Discussion

Brain tumors (BTs) are a heterogeneous group of neoplasms with unique biology, treatment, and prognosis (43). The treatment of BTs is challenging due to these cancers’ anatomic position and intrinsic factors, leading to high mortality and morbidity worldwide (44). The current focus of cancer research is to understand the genetic differences between the malignant cell to reach particular and effective therapy strategies (43). Brain and testis have many similarities since both glial cells and melanocytes are derived from the neuroectoderm. These two cell types share many biological and morphological features. The former can be referred to as a blood barrier and the latter is the gene whose expression is limited to these two tissues (45).

Many human neoplastic tissues express cancer-testis (CT) genes. The CT genes are a group of genes with different functions in normal germ cells, some of which are critical in the tumorigenesis process. CT gene expressions are restricted in normal tissues except in testis and a wide range of tumor types (10, 46). The expression of many cancer-testis (CT) genes in undifferentiated and differentiated BTs may provide particular targets for treating cancer recurrence and metastasis (45). Nevertheless, little is known about the function and different transcript expression patterns of the CT genes.

This study aimed to investigate the expression and transcription patterns of two potential cancer-testis genes, that is, *TSGA10* and *GGNBP2*. The expression of these genes was examined in different stages and grades of BTs, and the results revealed that these genes were highly expressed in the lower grades of BTs. The more the grade of the tumor increases, the more its expression decreases, meaning these two genes may act as tumor suppressor genes in these tumors.

We examined the mRNA expression profiles of *TSGA10* and *GGNBP2* transcript variants in BTs of different malignancy grades. Compared to the other tumor groups, high expressions of *TSGA10* and *GGNBP2* were observed in astrocytoma’s grade 2. Higher-grade BTs display a sharp decrease in *TSGA10* and *GGNBP2* transcripts, especially in medulloblastoma grade IV, given that *TSGA10* and *GGNBP2* expressions decrease in higher grades of BTs (**Table 4**). These genes may play a critical role in the development of BTs. Apart from that, the splicing of exons in the 5’UTR region and expression of transcripts with shorter 5’UTR sequences could decrease the translation efficiency of these genes and increase angiogenesis and the development of BTs in higher grades, especially in astrocytoma (42). The findings suggest that the *TSGA10* and *GGNBP2* transcript variants might present a functional role, altering the expression patterns of these genes, thereby increasing tumor growth and stimulating malignancy traits. Accordingly, understanding their expression levels may be informative relative to the influence of the *TSGA10* and *GGNBP2* genes in astrocytoma and, consequently, in patients’ prognoses.

**Table 4:** *TSGA10* and *GGNBP2* gene variants expression in 30 brain tumor samples.

Brain tumors samples	*TSGA10* F6R6 All Variants	*TSGA10* F1R3 KY607423	*TSGA10* F1R4 KY607424	*TSGA10* F4R3 KY607425	*TSGA10* F4R4 KY607426	GGNBP2 F1R1 All variants	GGNBP2 with exon6 F1R2	GGNBP2 without exon6 F1R2	GGNBP2 with exon2 F2R1	GGNBP2 without exon2 F2R1
**Schwannoma Grade I N=3**	4.26	3.9	3.3	3.64	1.23	3.93	2.9	4.1	3.1	4.1
**Medulloblastoma Grade IV N=5**	2.77	3.5	2.68	3.2	0.78	2.1	1.14	1.8	1.22	2.26
**GBM Grade IV N=9**	3.6	3.71	2.76	3.47	1.11	2.58	1.3	2.07	1.21	3.04
**Astrocytoma Grade II N=13**	3.86	3.99	3.05	3.77	1.37	3.56	2.71	3.6	2.8	3.75

GGNBP2 is primarily expressed in spermatogenesis and male germ cells and is necessary for testis morphology and sperm development (47). *GGNBP2* gene expression produces a protein binding to testicular germ-specific protein gametogenetin 1 (GGN1) (48). This protein is expressed in late pachytene in spermatocytes, simultaneous with meiosis, and is up-regulated in round spermatids. According to previous studies, GGNBP2 plays a crucial role in pregnancy prosperity by preserving equivalence in the proliferation and differentiation of trophoblast stem cells during placental progression (49). The overexpression of GGNBP2 decreases tumor cell proliferation, migration, invasion, angiogenesis, and metastasis of human cancers and arrest in the G1 phase of the cell cycle (22, 50). It down-regulates the protein levels of p-PI3K, p-Akt, Wnt, and b-catenin. This effect is significant since the activation of Wnt/b-catenin and PI3K/Akt signaling pathways results in the malignant development of various tumors, such as glioma (22). Accordingly, GGNBP2 acted as a tumor suppressor in patients with glioma and was suggested as a potential therapeutic method (50, 51).

**Figure 6:**
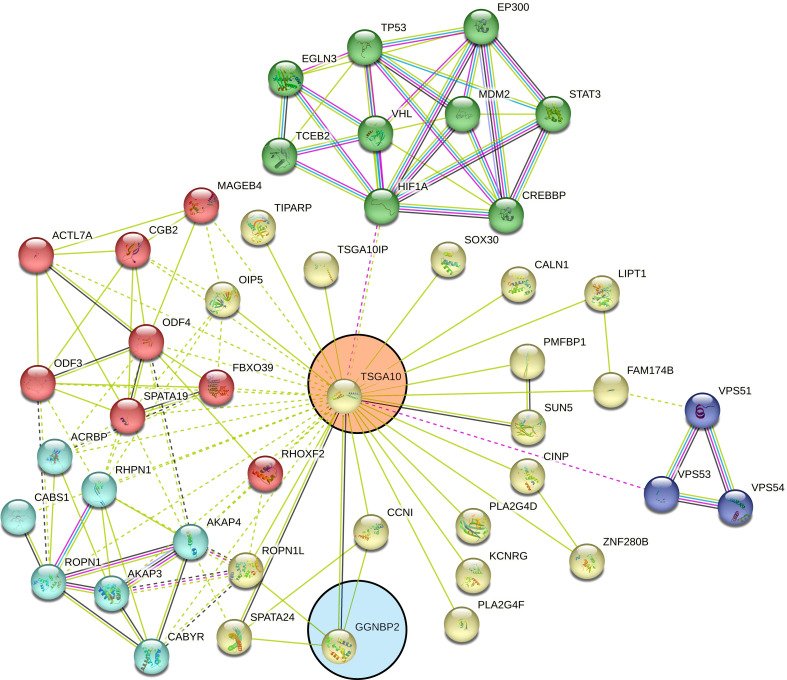
Clustered pathways of the *TSGA10* gene by string database. The network of protein–protein interactions (PPI) was constructed for the *TSGA10* gene, using the STRING online database. The genes were clustered based on the KEGG and the Reactome pathway databases. We selected the network modules related to the brain from the set of all TSGA10 networks. The genes were clustered based on the KEGG and the Reactome pathway databases. Networks were shown related to TSGA10 (in the pink circle) and GGNBP2 (in the blue circle) genes. Each line represents a STRING interaction score of greater than or equal to.

**Figure 7:**
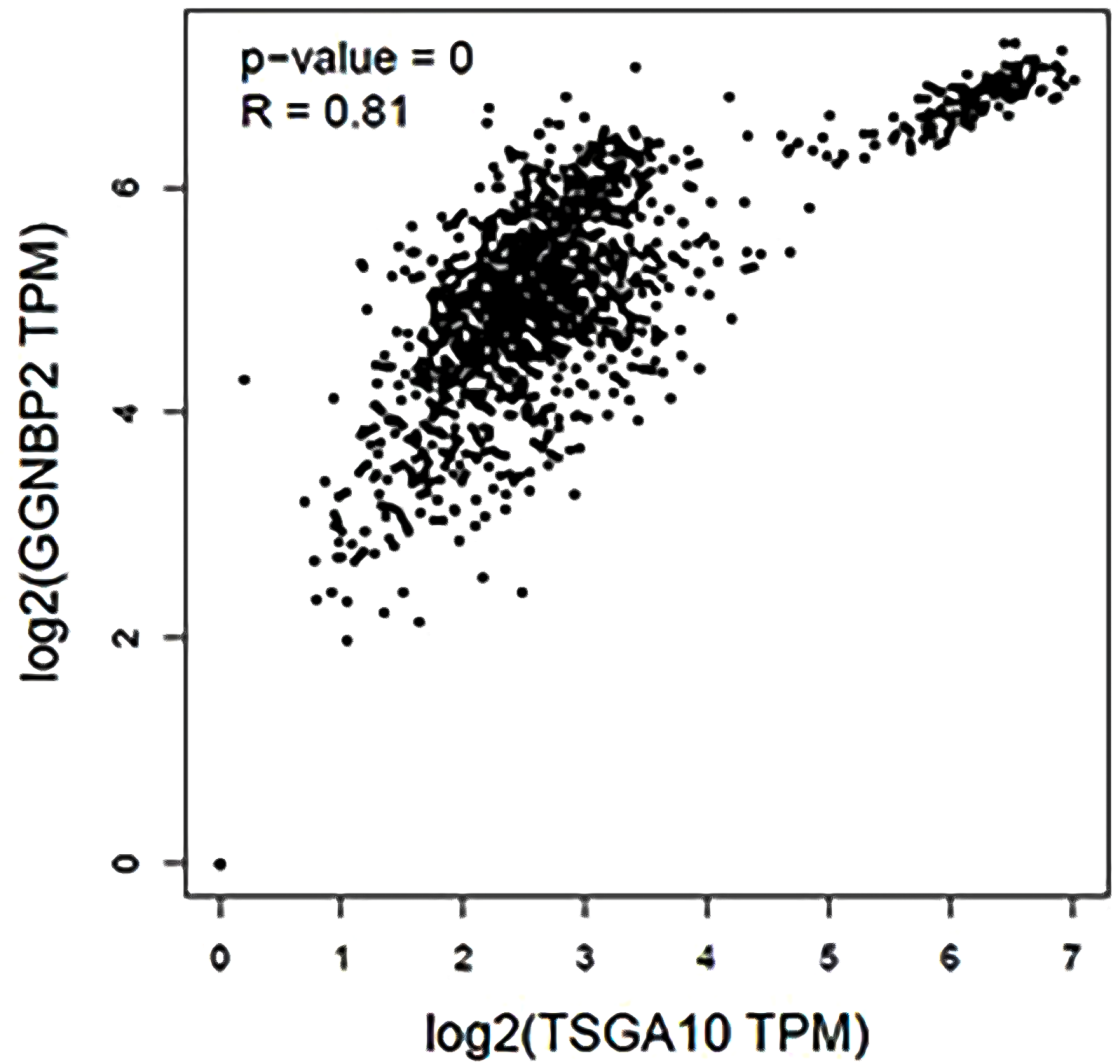
The expression correlation between *GGNBP2* and *TSGA10* genes that were shown by the GEPIA database. Co-expression results of genes predicted by the GEPIA database online analysis showed that there is a statistically significant spearman correlation between TSGA10 and GGNBP2 mRNA expression in BT and normal brain and testis based on TCGA (the cancer genome atlas) and GTEx (The Genotype-Tissue Expression) samples (Log-rank test; P=0 and R=0.81).

TSGA10 is primarily detected in the testis and plays key roles in various biological processes such as spermatogenesis, embryogenesis, angiogenesis, and malignancy (52–54). It was also revealed that TSGA10 is overexpressed in BTs (13). The over-expression of TSGA10 was associated with tumor suppression and cancerous cell apoptosis (55). Moreover, the *TSGA10* gene could express different transcript variants with the same coding region but different 5′UTR sequences in breast cancer (42). The pathway of the *TSGA10* gene was Depicted by using the string database. Then, we identified and selected the genes involved in the brain through clustering (**Figure 6**). Using the GEPIA database, we measured the correlation between the expression of each gene and *TSGA10* and observed that the *GGNBP2* gene has a high correlation with *TSGA10*. In addition, in the GEPIA database, TSGA10 and *GGNBP2* revealed a correlation expression of 81% in all brain tumor and normal brain and testis samples (**Figure 7**). In this study, these two genes revealed a correlation of 78.1% in 30 samples of BTs (**Table 5**).

**Table 5:** Correlations between the *TSGA10* and *GGNBP2* genes.

			GGNBP2
Spearman’s rho	TSGA10	Correlation Coefficient	781^**^
		Sig. (2-tailed)	000
		N	30

**. Significant at p= 0.01 (2-tailed).

Although the analysis of the microarray data for various BTs revealed significant changes in the expression of *GGNBP2* and *TSGA10* in BTs, these genes do not show drastic changes in all types of BTs. Accordingly, this might be associated with the alternative splicing of these genes in different tissues. This study showed that the human *TSGA10* gene consists of at least 22 exons, and the human *GGNBP2* gene consists of at least 14 exons, of which the first seven exons of *TSGA10* and the first two exons of *GGNBP2* form the 5′UTR region. The *TSGA10* gene can produce four transcript variants derived from two distinct promoter regions and differential splicing of exon 4. The *GGNBP2* gene can produce seven transcript variants due to exon splicing; two of the splicing exons are exon 2 splicing in the 5′UTR region and exon 6 splicing in the coding region. Regarding the transcript expression patterns, the relative mRNA expression of *TSGA10* and *GGNBP2* genes in the BT samples differed from normal testicular tissue; accordingly, the transcripts have a shorter 5′UTR sequence in tumor samples. In this study, for the first time, the expression mRNA levels of *TSGA10* and *GGNBP2* genes were investigated based on the splicing of their exons in BT samples. It was shown that the transcripts with longer 5’UTR have lower expression in BT samples, which can affect the translation ability of these genes (**Figures 4**, **5**).

Although our study showed the expression of different transcript variants of *TSGA10* and *GGNBP2* genes in brain tumor samples, several limitations could affect this study. One of the limitations of this study was the small number of examined samples in different types of brain tumors as well as in different types of stages and grades. In addition, the gold standard for determining the role of the 5′UTR region on the efficiency of protein synthesis is the Luciferase assay method, which was not performed in this study due to existing limitations.

## Conclusion

The present study undertook a microarray analysis and found that differential expression of *TSGA10* and *GGNBP2* were deregulated in brain tumor samples. We used RT-PCR to identify the expression pattern of *TSGA10* and *GGNBP2* transcript variants and the characteristics of their expression profiles in different brain tumor samples. We identified that the *TSGA10* and *GGNBP2* were expressed in all of the brain tumor and testicular tissue samples, but the expression of the transcript variants with longer 5′UTR was reduced in brain tumor samples, unlike the testis sample. The expression analysis results of the TSGA10 transcript variants in brain tumor samples were shown to have a higher expression of transcripts without exon 4 than transcripts with exon 4. The relative mRNA expression of the GGNBP2 transcript variants also showed that the expression of transcripts without exon 2 was higher than transcripts with exon 2. Downregulation of the transcript variants with longer 5′UTR could reduce the translation efficiency of these genes and this may lead to angiogenesis and progression of the brain tumor in patients.

## Data availability statement

The datasets presented in this study can be found in online repositories. The names of the repository/repositories and accession number(s) can be found in the article/**Supplementary Material**.

## Ethics statement

The studies involving human participants were reviewed and approved by Shahid Beheshti University of Medical Science Functional Neurosurgery research center [IR.SBMU.RETECH.REC.1399.1144]. Written informed consent to participate in this study was provided by the participants’ legal guardian/next of kin.

## Author contributions

RK, MHM, and PS designed the study. MSM and RK collected the samples and reviewed cases. RK and PS performed the experiments. RK and EA analyzed the data statistically. RK, EA, MSM, and MHM interpreted the results. RK and EA wrote the manuscript. MSM and MHM revised the manuscript. All authors have read and approved the final manuscript.
