# Supplementary material for: Identification of TSGA10 and GGNBP2 splicing variants in 5′ untranslated region with distinct expression profiles in brain tumor samples
Source: Front Oncol. 2023 Feb 13;13:1075638. doi: 10.3389/fonc.2023.1075638 (PMC9968883; doi:10.3389/fonc.2023.1075638)
Supplement: Supplementary file 1 [file DataSheet_1.docx]

**Supplemental tables and figures**

**Investigating expressed transcript variants of TSGA10 and GGNBP2 as a cancer‐testis antigen in brain tumor samples**

Reihane Kazerani, Pouya Salehipour, Mohammadreza Shah Mohammadi, Elnaz Amanzadeh Jajin, Mohammad Hossein Modarressi

**
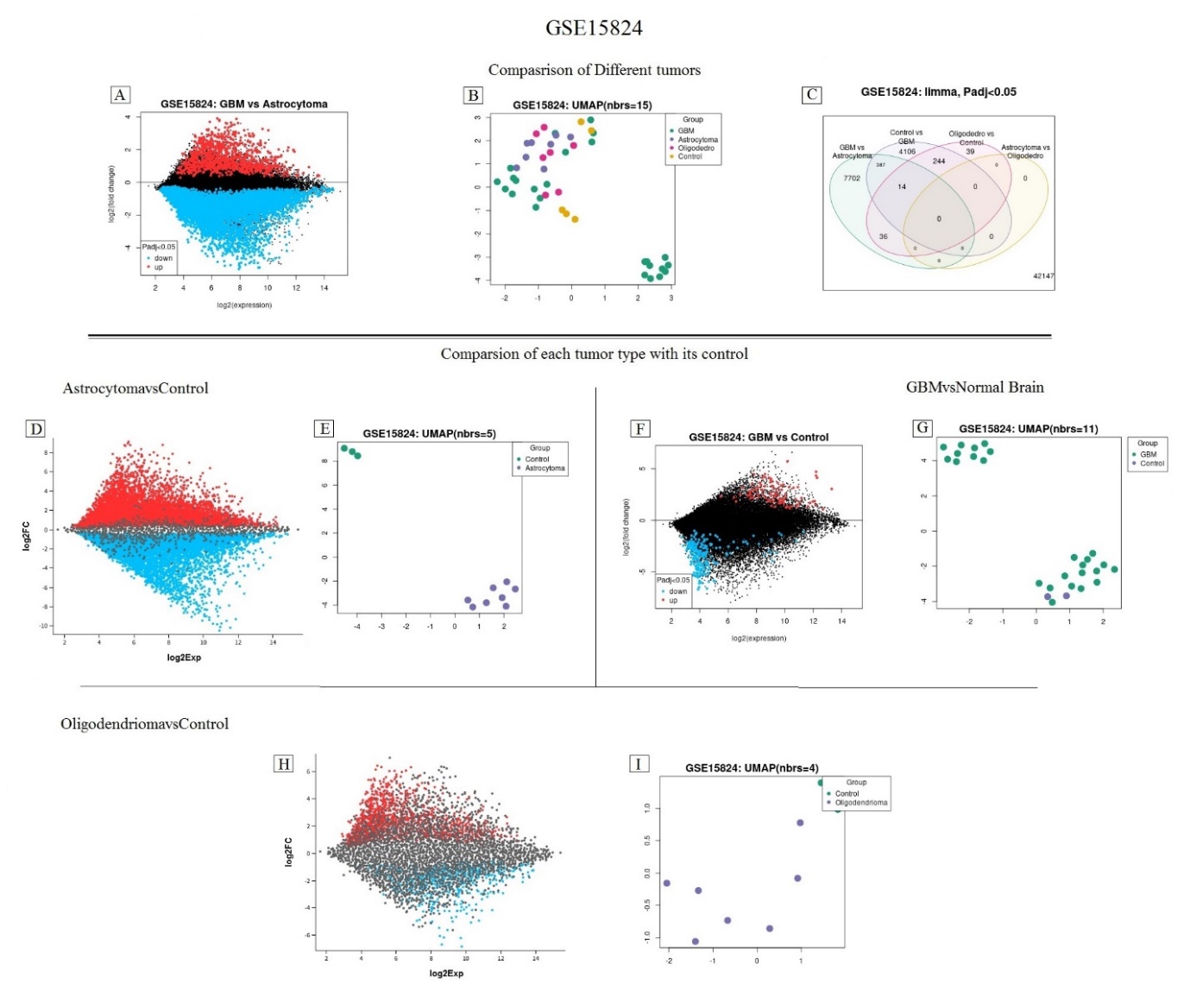
**

**Fig. S1** GSE15824 samples, including 30 BT samples (12 primary glioblastomas (GBM), 3 secondary glioblastomas (GBM-2), 8 astrocytomas and 7 oligodendrogliomas) and 5 glioblastoma cell lines (LN018, LN215, LN229, LN319 and BS149). Normal brain tissue (NB) and normal human astrocytes (NHA) were used as a control. Red dots represent significantly upregulated genes in BTs. Blue dots are significantly downregulated genes in BTs. DEGs were filtered based on LogFC over than 2 or less than −2 with an adjusted p-value set as 0.05. (A) Volcano plots comparing gene expression between GBM and astrocytoma. (B) The UMAP algorithm classifies BTs into four clusters indicated by different colors. UMAP projection of microarray data comparing BTs and NB. (C) A Venn diagram of DEGs in BTs versus normal samples. (D) Volcano plots comparing gene expression between astrocytoma and NB. DEGs were filtered based on LogFC over than 2 or less than −2 with an adjusted p-value set as 0.05. (E) The UMAP plot classifies astrocytoma and NB into two clusters indicated by different colors. (F) Volcano plots comparing gene expression between GBM and NB. (G) The UMAP plot classifies GBM and NB into two clusters indicated by different colors. The UMAP plot classifies Medulloblastoma and control into two clusters indicated by different colors. (H) Volcano plots comparing gene expression between oligodendrioma and NB. (I) The UMAP plot classifies oligodendrioma and NB into two clusters indicated by different colors.

**
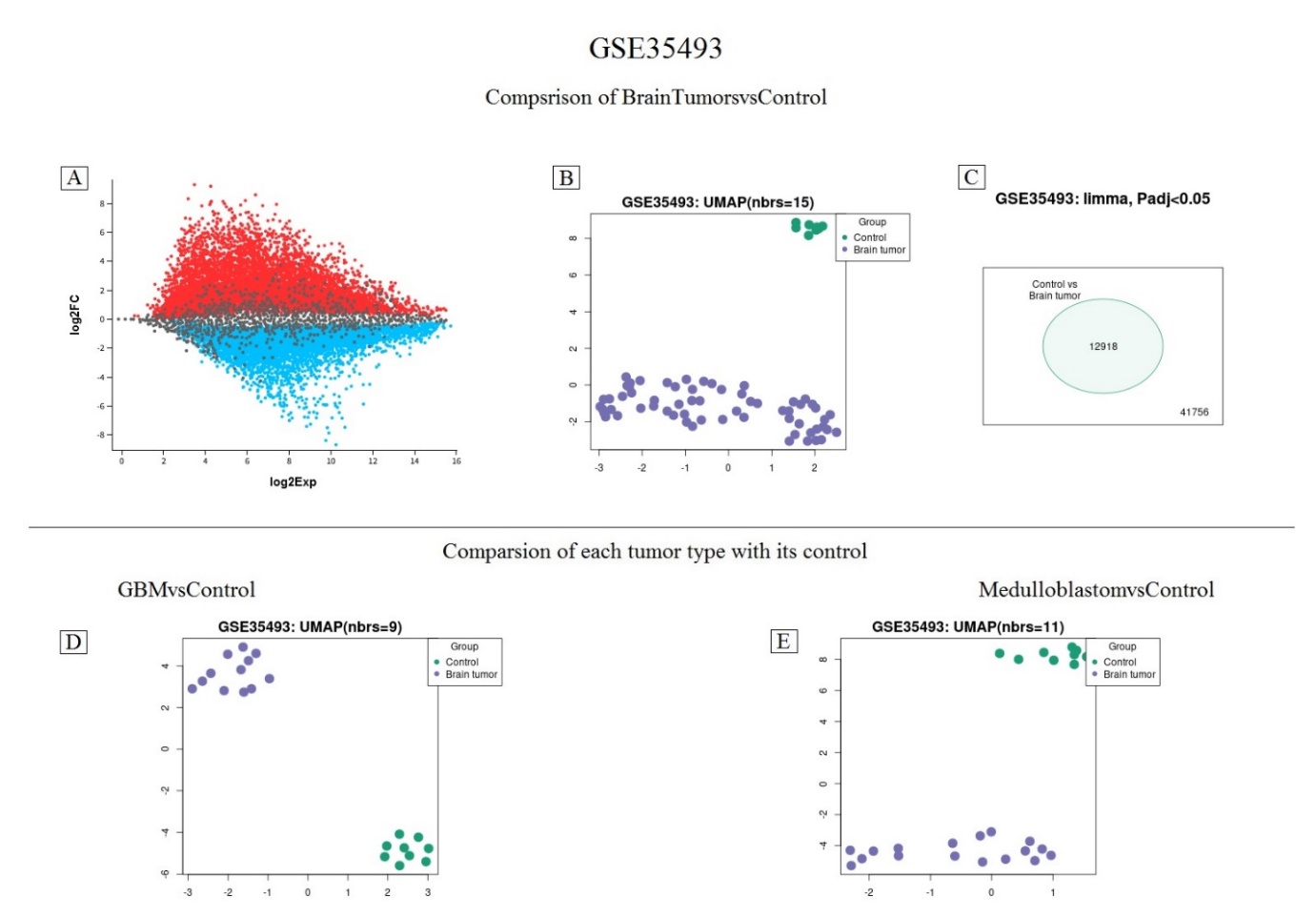
**

**Fig. S2** GSE35493 samples, including20 ATRTs, 42 other pediatric CNS Grade IV tumor samples, and 9 pediatric NB samples, using Affymetrix U133 Plus2 GeneChips. (A)The resulting hazard ratios (HR) were visualized on a volcano plot, with the indicated top hits. Volcano plots comparing gene expression between BTs and control samples. Red dots represent significantly upregulated genes in BTs. Blue dots are significantly downregulated genes in BTs. DEGs were filtered based on LogFC over than 2 or less than −2 with an adjusted p-value set as 0.05. (B) The UMAP algorithm classifies BTs into two clusters indicated by different colors. UMAP projection of microarray data comparing BTs and NB. Hierarchical clustering of top 1000 variable genes from BTs and NB. (C) A Venn diagram of DEGs in BTs versus normal samples. Genes with false discovery rate <0.05 were considered to be differentially expressed. (D) The UMAP plot classifies GBM and control into two clusters indicated by different colors. (E) The UMAP plot classifies Medulloblastoma and control into two clusters indicated by different colors.

**
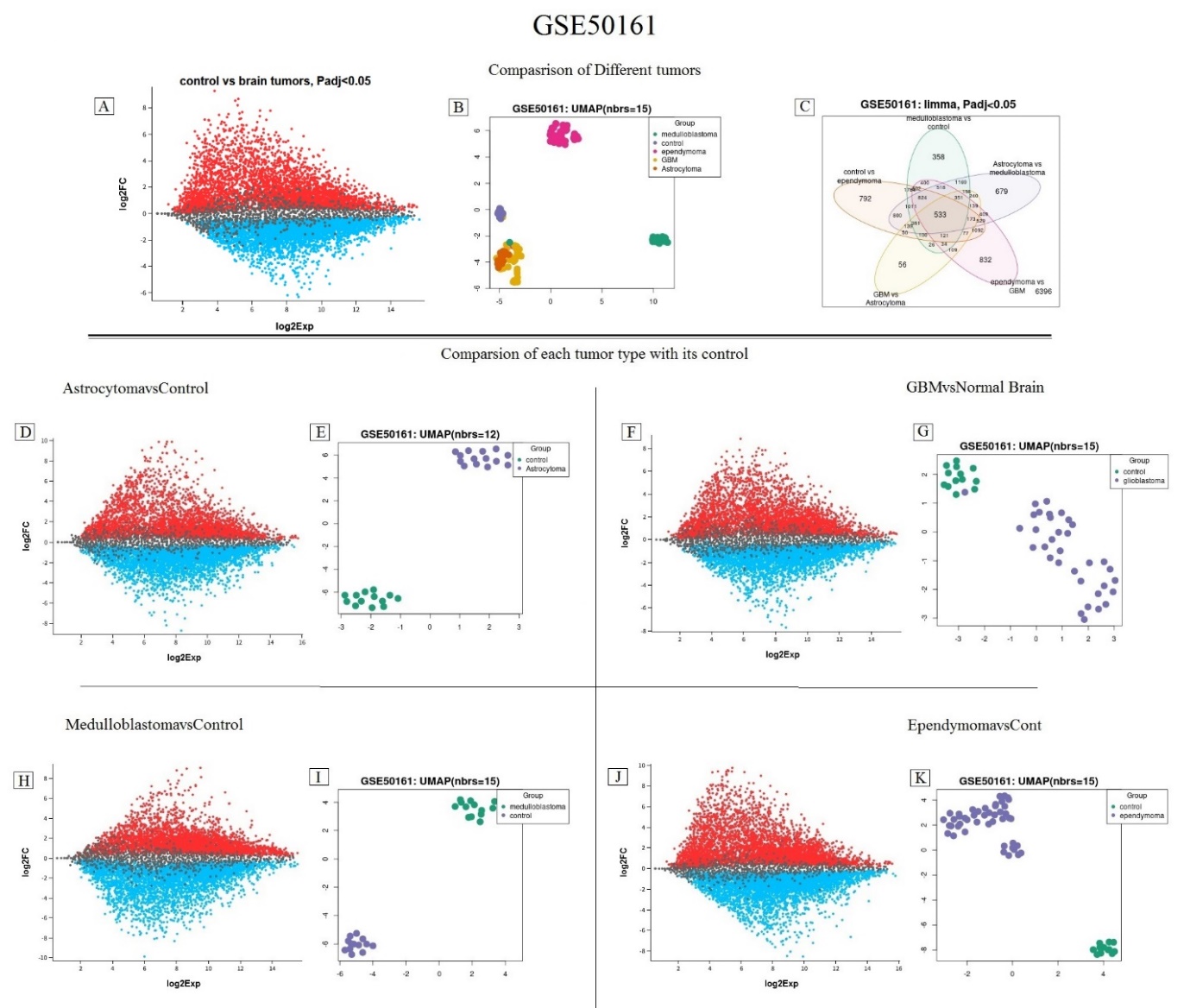
**

**Fig. S3** GSE50161 samples (n=130), including surgical tumor (ependimoma (n=46), GBM (n=34), medollublastoma (n=22), astrocytoma (n=15)) and NB (n=13), using Affymetrix HG-U133plus2 chips (Platform GPL570). Blue dots are significantly downregulated genes in BTs. DEGs were filtered based on Log FC over than 2 or less than −2 with an adjusted p-value set as 0.05. (A) Volcano plots comparing gene expression between BTs and NB. Red dots represent significantly upregulated genes in BTs. (B) The UMAP algorithm classifies BTs into five clusters indicated by different colors. UMAP projection of microarray data comparing BTs and NB. (C) A Venn diagram of DEGs in BTs and NB samples. (D) Volcano plots comparing gene expression between astrocytoma and NB. (E) The UMAP plot classifies astrocytoma and NB into two clusters indicated by different colors. (F) Volcano plots comparing gene expression between GBM and NB. (G) The UMAP plot classifies GBM and NB into two clusters indicated by different colors. The UMAP plot classifies GBM and control into two clusters indicated by different colors. (H) Volcano plots comparing gene expression between medulloblastoma and NB. (I) The UMAP plot classifies medulloblastoma and NB into two clusters indicated by different colors. (J) Volcano plots comparing gene expression between ependimoma and NB. (K) The UMAP plot classifies ependimoma and NB into two clusters indicated by different colors.

**Table S1.** Results of DEG analysis for TSGA10 and GGNBP2 in each dataset.

| series | ID | adj.P.Val | P.Value | t | B | logFC | Gene.symbol | Gene.title |
| --- | --- | --- | --- | --- | --- | --- | --- | --- |
| GSE15824 | 220623_s_at | 9.14E-01 | 3.02E-01 | -1.04 | -5.647053 | -0.63875597 | TSGA10 | testis specific 10 |
| GSE15824 | 1555931_at | 9.24E-01 | 5.08E-01 | 6.67E-01 | -5.957004 | 0.35242567 | TSGA10 | testis specific 10 |
| GSE15824 | 1555932_at | 9.38E-01 | 6.95E-01 | -3.95E-01 | -6.097761 | -0.25997972 | TSGA10 | testis specific 10 |
| GSE15824 | 223838_at | 9.69E-01 | 8.49E-01 | -1.91E-01 | -6.156079 | -0.13772381 | TSGA10 | testis specific 10 |
| GSE15824 | 233936_s_at | 9.15E-01 | 3.04E-01 | -1.04 | -5.650016 | -0.4794312 | GGNBP2 | gametogenetin binding protein 2 |
| GSE15824 | 1554419_x_at | 9.19E-01 | 3.24E-01 | -9.98E-01 | -5.691403 | -0.44730869 | GGNBP2 | gametogenetin binding protein 2 |
| GSE15824 | 218079_s_at | 9.30E-01 | 6.19E-01 | -5.01E-01 | -6.051243 | -0.22300693 | GGNBP2 | gametogenetin binding protein 2 |
| GSE50161 | 223838_at | 7.42E-01 | 6.15E-01 | 0.506957 | -6.848685 | 0.1443747 | TSGA10 | testis specific 10 |
| GSE50161 | 233936_s_at | 2.70E-01 | 1.46E-01 | -1.477211 | -5.898803 | -0.3872093 | GGNBP2 | gametogenetin binding protein 2 |
| GSE35493 | 1555931_at | 3.41E-01 | 2.21E-01 | 1.2642871 | -6.320468 | 0.05453939 | TSGA10 | testis specific 10 |
| GSE35493 | 220623_s_at | 3.59E-01 | 2.43E-01 | -1.2021567 | -6.394722 | -0.23538737 | TSGA10 | testis specific 10 |
| GSE35493 | 1555932_at | 4.65E-01 | 3.74E-01 | 0.9097667 | -6.699441 | 0.12653388 | TSGA10 | testis specific 10 |
| GSE35493 | 223838_at | 6.54E-01 | 5.81E-01 | -0.560414 | -6.959885 | -0.08635155 | TSGA10 | testis specific 10 |
| GSE35493 | 233937_at | 6.09E-01 | 5.32E-01 | -0.6362491 | -6.91339 | -0.08524104 | GGNBP2 | gametogenetin binding protein 2 |
| GSE35493 | 233936_s_at | 8.26E-01 | 7.80E-01 | 0.2825575 | -7.08086 | 0.07859686 | GGNBP2 | gametogenetin binding protein 2 |
| GSE35493 | 218079_s_at | 1.12E-02 | 1.92E-03 | -3.5690445 | -1.988467 | -0.88123036 | GGNBP2 | gametogenetin binding protein 2 |
| GSE35493 | 1554419_x_at | 2.18E-01 | 9.13E-02 | 1.7734628 | -5.5993 | 0.21006785 | GGNBP2 | gametogenetin binding protein 2 |
| GSE15824 | 220623_s_at | 0.02509 | 7.34E-05 | -5.5697559 | 1.86638 | -2.1176846 | TSGA10 | testis specific 10 |
| GSE15824 | 223838_at | 0.10453 | 3.49E-03 | -3.5177963 | -1.72498 | -2.1694464 | TSGA10 | testis specific 10 |
| GSE15824 | 1555932_at | 0.48007 | 1.54E-01 | -1.5101797 | -5.11133 | -1.0752796 | TSGA10 | testis specific 10 |
| GSE15824 | 1555931_at | 0.98643 | 9.61E-01 | -0.0502039 | -6.19196 | -0.0285658 | TSGA10 | testis specific 10 |

**Table S2.** Results of DEG analysis for TSGA10 and GGNBP2 in each dataset.

|  | ID | adj.P.Val | P.Value | T | B | logFC | Gene symbol |
| --- | --- | --- | --- | --- | --- | --- | --- |
| GSE15824.comparison of different tumors | 220623_s_at | 1.46E-02 | 1.97E-04 | -4.0514790 | 0.620023 | -1.39 | TSGA10 |
| GSE15824.comparison of different tumors | 223838_at | 1.09E-01 | 5.25E-03 | -2.9332280 | -2.355676 | -1.25 | TSGA10 |
| GSE15824.comparison of different tumors | 1555932_at | 2.88E-01 | 3.79E-02 | -2.1380259 | -4.074318 | -8.52E-01 | TSGA10 |
| GSE15824.comparison of different tumors | 1555931_at | 9.37E-01 | 8.71E-01 | -0.1636881 | -6.193958 | -5.50E-02 | TSGA10 |
| GSE15824.comparison of different tumors | 1554419_x_at | 4.82E-01 | 1.58E-01 | -1.4345795 | -5.220385 | -4.04E-01 | GGNBP2 |
| GSE15824.comparison of different tumors | 233936_s_at | 5.18E-01 | 2.01E-01 | -1.2977886 | -5.396229 | -4.03E-01 | GGNBP2 |
| GSE15824.comparison of different tumors | 233937_at | 6.59E-01 | 4.09E-01 | -0.8340078 | -5.868485 | -3.13E-01 | GGNBP2 |
| GSE15824.comparison of different tumors | 218079_s_at | 8.54E-01 | 7.22E-01 | -0.3582671 | -6.144211 | -1.03E-01 | GGNBP2 |
| GSE15824.AstrocytomavsCon | 1555931_at | 5.88E-02 | 1.78E-02 | -2.78 | -4.4690775 | -6.29E-01 | TSGA10 |
| GSE15824.AstrocytomavsCon | 233936_s_at | 5.41E-02 | 1.60E-02 | 2.83 | -4.3648674 | 2.95E-01 | GGNBP2 |
| GSE15824.GBMvsCon | 220623_s_at | 0.032296 | 8.31E-05 | -4.616008 | 1.51741 | -2.5957911 | TSGA10 |
| GSE15824.GBMvsCon | 1554419_x_at | 0.568168 | 2.41E-01 | -1.1991115 | -5.31981 | -0.5237986 | GGNBP2 |
| GSE15824.OligodendrivsCon | 223838_at | 0.001821 | 3.76E-06 | 10.1708838 | 4.957538 | 2.5217432 | TSGA10 |
| GSE15824.OligodendrivsCon | 1554419_x_at | 0.493671 | 1.31E-01 | -1.6650746 | -5.358804 | -0.4170799 | GGNBP2 |
| GSE50161.comparison of different tumors | 223838_at | 1.20E-15 | 1.10E-16 |  |  | 28.07277 | TSGA10 |
| GSE50161.comparison of different tumors | 233936_s_at | 1.51E-01 | 1.12E-01 |  |  | 1.91756 | GGNBP2 |
| GSE50161.AstrovsCon | 223838_at | 4.74E-01 | 2.88E-01 | 1.0839798 | -6.708429 | 0.22623726 | TSGA10 |
| GSE50161.AstrovsCon | 233936_s_at | 9.14E-01 | 8.49E-01 | -0.1927854 | -7.282855 | -0.0446718 | GGNBP2 |
| GSE50161.GBMvsCon | 223838_at | 7.42E-01 | 6.15E-01 | -0.506957 | -6.848685 | -0.1443747 | TSGA10 |
| GSE50161.GBMvsCon | 233936_s_at | 2.70E-01 | 1.46E-01 | 1.477211 | -5.898803 | 0.3872093 | GGNBP2 |
| GSE50161.medullovsCon | 223838_at | 7.24E-01 | 5.51E-01 | -0.6015944 | -7.105337 | -0.137477 | TSGA10 |
| GSE50161.medullovsCon | 233936_s_at | 8.88E-01 | 7.69E-01 | -0.2955998 | -7.24506 | -0.0758701 | GGNBP2 |
| GSE50161.ependymomavsCon | 223838_at | 1.48E-04 | 3.22E-05 | -4.5076014 | 1.28574 | -2.6775173 | TSGA10 |
| GSE50161.ependymomavsCon | 233936_s_at | 9.06E-01 | 8.28E-01 | -0.2187246 | -7.52624 | -0.0463902 | GGNBP2 |
| GSE35493.comparison of different tumors | 220623_s_at | 9.90E-01 | 9.86E-01 | -0.017284 | -7.1065386 | -0.0049009 | TSGA10 |
| GSE35493.comparison of different tumors | 1555931_at | 3.32E-01 | 1.79E-01 | -1.358895 | -6.1885058 | -0.0447958 | TSGA10 |
| GSE35493.comparison of different tumors | 1555932_at | 5.48E-01 | 4.50E-01 | -0.760014 | -6.816885 | -0.0809682 | TSGA10 |
| GSE35493.comparison of different tumors | 223838_at | 5.95E-01 | 5.05E-01 | -0.670507 | -6.8809195 | -0.2235875 | TSGA10 |
| GSE35493.comparison of different tumors | 233936_s_at | 7.94E-01 | 7.38E-01 | -0.335172 | -7.0501377 | -0.0836601 | GGNBP2 |
| GSE35493.comparison of different tumors | 233937_at | 4.76E-01 | 3.67E-01 | -0.908423 | -6.6933835 | -0.2380426 | GGNBP2 |
| GSE35493.comparison of different tumors | 1554419_x_at | 2.04E-01 | 6.98E-02 | -1.841653 | -5.4383037 | -0.1833831 | GGNBP2 |
| GSE35493.comparison of different tumors | 218079_s_at | 3.21E-02 | 6.94E-03 | 2.781637 | -3.410511 | 0.5634092 | GGNBP2 |
| GSE35493.GBMvsCon | 1555931_at | 3.41E-01 | 2.21E-01 | -1.2642871 | -6.320468 | -0.0545393 | TSGA10 |
| GSE35493.GBMvsCon | 220623_s_at | 3.59E-01 | 2.43E-01 | 1.2021567 | -6.394722 | 0.23538737 | TSGA10 |
| GSE35493.GBMvsCon | 1555932_at | 4.65E-01 | 3.74E-01 | -0.9097667 | -6.699441 | -0.1265338 | TSGA10 |
| GSE35493.GBMvsCon | 223838_at | 6.54E-01 | 5.81E-01 | 0.560414 | -6.959885 | 0.08635155 | TSGA10 |
| GSE35493.GBMvsCon | 233936_s_at | 8.26E-01 | 7.80E-01 | -0.2825575 | -7.08086 | -0.0785968 | GGNBP2 |
| GSE35493.GBMvsCon | 218079_s_at | 1.12E-02 | 1.92E-03 | 3.5690445 | -1.988467 | 0.88123036 | GGNBP2 |
| GSE35493.GBMvsCon | 1554419_x_at | 2.18E-01 | 9.13E-02 | -1.7734628 | -5.5993 | -0.2100678 | GGNBP2 |
| GSE35493.GBMvsCon | 233937_at | 6.09E-01 | 5.32E-01 | 0.6362491 | -6.91339 | 0.08524104 | GGNBP2 |
| GSE35493.medullovsCon | 1555931_at | 4.15E-01 | 2.34E-01 | -1.2186887 | -6.458727 | -0.0383709 | TSGA10 |
| GSE35493.medullovsCon | 223838_at | 5.71E-01 | 4.68E-01 | -0.7365012 | -6.92726 | -0.2027232 | TSGA10 |
| GSE35493.medullovsCon | 1555932_at | 6.85E-01 | 6.05E-01 | -0.5243359 | -7.063378 | -0.0599832 | TSGA10 |
| GSE35493.medullovsCon | 220623_s_at | 9.67E-01 | 9.55E-01 | -0.056928 | -7.203115 | -0.0160799 | TSGA10 |
| GSE35493.medullovsCon | 233936_s_at | 8.18E-01 | 7.64E-01 | 0.3033551 | -7.157284 | 0.08980303 | GGNBP2 |
| GSE35493.medullovsCon | 218079_s_at | 3.99E-01 | 2.11E-01 | 1.2830597 | -6.380376 | 0.28135074 | GGNBP2 |
| GSE35493.medullovsCon | 233937_at | 4.09E-01 | 2.25E-01 | -1.2455108 | -6.426512 | -0.4292776 | GGNBP2 |
| GSE35493.medullovsCon | 1554419_x_at | 2.80E-01 | 1.12E-01 | -1.6491966 | -5.869911 | -0.1372213 | GGNBP2 |
